# Salivary melatonin in oral squamous cell carcinoma patients

**DOI:** 10.1038/s41598-021-92649-3

**Published:** 2021-06-24

**Authors:** Ivan Salarić, Ivana Karmelić, Jasna Lovrić, Ksenija Baždarić, Marko Rožman, Igor Čvrljević, Ivan Zajc, Davor Brajdić, Darko Macan

**Affiliations:** 1 Department of Oral Surgery, University of Zagreb School of Dental Medicine, Av. Gojka Šuška 6, 10000 Zagreb, Croatia; 2 Department of Maxillofacial and Oral Surgery, University Hospital Dubrava, Zagreb, Croatia; 3 Department of Medical Chemistry, Biochemistry and Clinical Chemistry, University of Zagreb School of Medicine, Zagreb, Croatia; 4 Department of Medical Informatics, Faculty of Medicine, University of Rijeka, Rijeka, Croatia; 5 Department of Physical Chemistry, Ruđer Bošković Institute, Zagreb, Croatia

**Keywords:** Cancer, Biomarkers

## Abstract

Melatonin’s role in circadian rhythm is well documented, as are its’ anti-oxidant, oncostatic and anti-inflammatory properties. Poor sleep quality has been associated as a potential risk factor for several malignancies, including head and neck cancers. The purpose of this study is to determine salivary melatonin (MLT) levels in oral squamous cell carcinoma (OSCC) patients, compare the salivary MLT levels with those in healthy individuals and compare the salivary and serum levels in OSCC patients. Furthermore, the aim is to investigate the potential relationship between sleep quality and salivary MLT levels in OSCC patients. Unstimulated (UWS) and stimulated (SWS) whole saliva was sampled from patients with T1N0M0 and T2N0M0 OSCC (N = 34) and 33 sex and age matched healthy subjects. Serum samples were taken from 11 OSCC patients. Sleep quality was measured using Pittsburgh Sleep Quality Index (PSQI) questionnaire. Melatonin levels in UWS and SWS were significantly higher in the OSCC group. Sleep quality was significantly lower in patients with OSCC (*P* = 0.0001). ROC analysis was found to be significant (*P* < 0.001) in evaluating MLT concentration limit in diagnosing OSCC. The expected relationship between sleep quality and salivary MLT levels in OSCC patients was not observed. Our results suggest salivary MLT as a potential biomarker that might facilitate non-invasive detection of early stage OSCC.

## Introduction

More than 325,000 people worldwide die from head and neck cancers every year^1,2^. Oral squamous cell carcinoma (OSCC) makes up nearly 55% of all head and neck cancers and has a 60% 5-year survival rate^1,2^. Tobacco and alcohol consumption are long known risk habits for OSCC development. Unfortunately, this disease is often diagnosed in the advanced stage and tissue histopathological examination is still considered as a gold standard for OSCC diagnosis. A number of studies identified potential salivary, tissue, plasma and serum biomarkers for OSCC, but unfortunately, none of the suggested biomarkers are in clinical use^3–6^. Apart from diagnostics, search for biomarkers may contribute towards a better understanding of OSCC carcinogenesis.

Recently, several cancer research studies focused on melatonin (*N*-acetyl-5-methoxytryptamine) (MLT) due to its’ anti-oxidant, oncostatic and anti-inflammatory properties^7–10^. Melatonin’s role in circadian rhythm is well documented and sleep deprivation has been associated with head and neck, breast, prostate and other cancers^11–13^. In vitro studies reported growth reduction in different types of cancer due to MLT oncostatic and anti-oxidant properties^14–16^. However, studies on serum MLT levels in different types of cancer patients have yielded conflicting results. Decreased serum MLT levels have been registered in patients with breast cancer, prostate cancer, lung cancer, stomach and colon cancer^17–20^. One of the suggested explanations for this was decreased sleep quality and increased fatigue in cancer patients^18,20^. Thereby, decreased sleep quality would result in the aberrant MLT synthesis during night-time and the overall MLT circadian rhythm. On the other hand, several papers have registered elevated serum MLT levels in colorectal carcinoma, melanoma and multiple myeloma^21–23^. These results have been explained by a reaction to cellular damage and a protective mechanism i.e. the overproduction of MLT for the purpose of stimulating the immune system and scavenging the free radicals. The exact mechanism and the role of the impaired MLT secretion in carcinogenesis of these malignancies remains unclear.

The role of MLT in OSCC has been investigated in several papers. Lu et al.^24^ and Yeh et al.^25^ showed that MLT represses OSCC metastasis. Yang et al. revealed the beneficial effect of MLT in the reduction of OSCC proliferation^26^. Stanciu et al.^27^ measured serum MLT levels in patients with OSCC and presented a clinical predictive model for the severity of OSCC. They obtained lower serum MLT levels in OSCC patients compared to healthy individuals and suggested a negative correlation between MLT serum levels and the severity of OSCC stages.

Due to its’ proximity to cancer cells and non-invasive sampling, saliva as a diagnostic fluid has its’ clear benefits in OSCC biomarker and carcinogenesis research. Furthermore, it is less expensive and more accessible than tissue, serum or plasma sampling. Recent studies found matrix metallopeptidase 2 (MMP2) and 9 (MMP9), Cyfra 21-1, tissue polypetide antigen (TPA), cancer antigen CA-125 and tumour necrosis factor α (TNF-α), interleukin 8 (IL-8) and IL-1β elevated in the saliva of OSCC patients^28,29^.

According to the available literature, salivary MLT levels in OSCC patients have not yet been measured. The aim of this research is to measure MLT in unstimulated whole saliva (UWS) and stimulated whole saliva (SWS) in OSCC patients and to compare the salivary and serum MLT levels in OSCC patients. Furthermore, the aim is to assess respondents’ sleep quality using the Pittsburgh Sleep Quality Index (PSQI) in order to observe the possible causal link with salivary MLT levels.

## Results

Thirty-four OSCC patients and 33 healthy individuals were included in this study, out of which 48 were male (71.6%) and 19 female (28.4%) (Table 1). There was no statistical difference in age (t = 0.793; *P* = 0.43) and sex (χ^2^ = 0.12, *P* = 0.730) distribution between groups. We report no missing data since none of the subjects had refused to participate or quit during the experiment and since all specimens were successfully analysed.

**Table 1:** Oral squamous cell carcinoma (OSCC) group and control group description.

	OSCC group (N = 34)	Control group (N = 33)	Statistics
Age (mean ± SD)	60.6 ± 11.1	63.0 ± 13.3	t = 0.793; *P* = 0.43
Sex (male (%)/female (%))	25 (73.53%)/9 (26.47%)	23 (69.70%)/10 (30.30%)	χ^2^ = 0.12; *P* = 0.73
T1N0M0	14 (41.18%)	–	–
T2N0M0	20 (58.82%)	–	–
Only smoked	5 (14.71%)	13 (39.40%)	χ^2^ = 5.12; *P* = 0.02
Only consumed alcohol	5 (14.71%)	5 (15.15%)	χ^2^ = 0.003; *P* = 0.96
Consumed alcohol and smoked	14 (41.18%)	1 (3.03%)	*P* = 0.0002
Alcohol altogether	Does not drink: 4 (11.76%) 1 a.u./day: 10 (29.41%) 2–4 a.u./day: 9 (26.47%) > 5a.u./day: 11 (32.35%)	Does not drink: 14 (42.42%) 1 a.u./day: 12 (36.36%) 2–4 a.u./day: 5 (15.15%) > 5a.u./day: 2 (6.06%)	χ^2^ = 13.10; *P* = 0.004
Smoking altogether	Does not smoke:14 (41.18%) 1–5 cig./day: 3 (8.82%) 6–10 cig./day: 1 (2.94%) 11–20 cig./day: 3 (8.82%) 21–35 cig./day: 9 (26.47%) > 36 cig./day: 4 (11.76%)	Does not smoke:15 (45.45%) 1–5 cig./day: 4 (12.12%) 6–10 cig./day: 8 (24.24%) 11–20 cig./day: 6 (18.18%) 21–35 cig./day: 0 (0%) > 36 cig./day: 0 (0%)	χ2 = 0.12; *P* = 0.73
No risk habits	10 (29.41%)	14 (42.42%)	χ^2^ = 1.23; *P* = 0.27

^a^*OSCC* oral squamous cell carcinoma, *SD* standard deviation, *a.u*. alcohol unit, *cig*. cigarettes.

Alcohol consumption between groups differed significantly (χ^2^ = 13.10; *P* = 0.004). More patients in the experimental group consumed over 5 alcohol units than those in the control group (t test proportion *P* = 0.046). The distribution of respondents with respect to tobacco consumption did not differ significantly between groups (χ^2^ = 0.12; *P* = 0.73). However, a significantly higher number of cigarettes was consumed by the experimental group (OSCC) (χ^2^ = 19.61; *P* = 0.0015) (Table 1).

Most common OSCC localisation was the body and the apex of the tongue (N = 16 (47.06%)), followed by the sublingual region and the mandibular gingiva (both N = 5 (14.71%)), retromolar region in the lower jaw and the maxillary gingiva (both N = 3 (8.82%)), cheek and the hard palate (both N = 1 (2.94%)). Twelve out of 16 tongue OSCC were staged as T2N0M0, along with 3 out of 5 sublingual, two out of 5 located in the mandibular gingiva, one out of 3 in the retromolar region of the mandible and maxillary gingiva and the one on the hard palate. The rest of the OSCC were T1N0M0.

**Figure 1:**
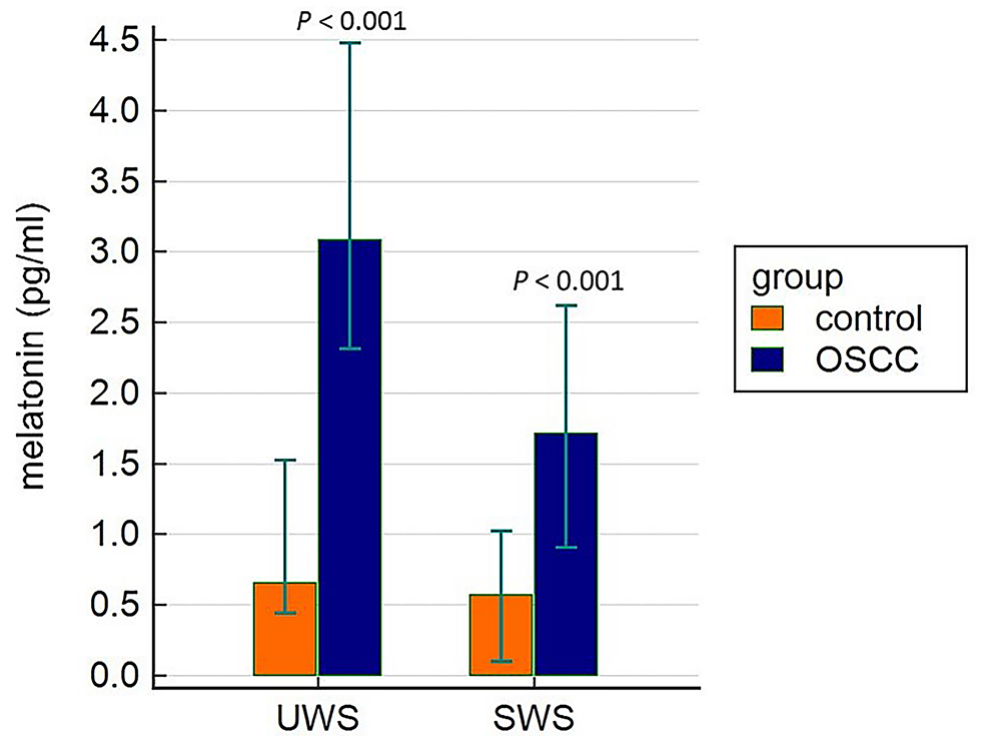
Comparison of salivary melatonin levels (pg/ml) in unstimulated whole saliva (UWS) and stimulated whole saliva (SWS) between the oral squamous cell carcinoma (OSCC) and the control group expressed with median and 95% confidence interval for the median.

Melatonin concentrations in UWS and SWS were significantly higher in the OSCC group compared to the control group (Table 2; Fig. 1). Furthermore, MLT levels were higher in the UWS than in SWS both in the control [Mann Whitney’s U test value (U) = 178.50; *P* < 0.0001] and the OSCC group (U = 263.50; *P* = 0.0002). When observing the OSCC subgroups, significant difference was found between patients with T1N0MO and T2N0M0 OSCC and the control group in the UWS and SWS (Table 3).

**Table 2:** Melatonin levels comparison in unstimulated and stimulated whole saliva between the groups and descriptive statistics for melatonin serum values in oral squamous cell carcinoma patients.

	OSCC group (N = 34)	Control group (N = 33)	OSCC group (N = 34)	Control group (N = 33)	OSCC group (N = 11)
	UWS		SWS		SERUM
Minimal value (pg/ml)	0.52	0.10^b=2^	0.10^b=1^	0.10^b=5^	6.16
Maximal value (pg/ml)	18.98	6.31	13.91	3.40	27.16
Median (95% CI)	3.08 (2.31–4.47)	0.66 (0.44–1.52)	1.71 (0.91–2.61)	0.57 (0.10–1.02)	13.01 (10.08–15.14)
Interquartile range	1.71–4.97	0.35–1.80	0.85–4.53	0.10–1.27	–
Statistics	U = 178.50	*P* < 0.001	U = 263.50	*P* < 0.001	–

^a^*OSCC* oral squamous cell carcinoma, *CI* confidence interval, *UWS* unstimulated whole saliva, *SWS* stimulated whole saliva.

^b^Number of values below the limit of detection.

**Table 3:** Melatonin levels comparison in unstimulated and stimulated whole saliva between oral squamous cell carcinoma subgroups and control group.

	T1N0M0 group (N = 14)	Control group (N = 33)	T1N0M0 group (N = 14)	Control group (N = 33)	T2N0M0 group (N = 20)	Control group (N = 33)	T2N0M0 group (N = 20)	Control group (N = 33)
	UWS		SWS		UWS		SWS	
Minimal value (pg/ml)	1.12	0.10^b=2^	0.10	0.10^b=5^	0.52	0.10^b=2^	0.10^b=1^	0.10^b=5^
Maximal value (pg/ml)	10.71	6.31	13.91	3.40	18.98	6.31	13.54	3.40
Median (95% CI)	3.67 (2.36–5.29)	0.66 (0.44–1.52)	2.42 (0.86–6.22)	0.57 (0.10–1.02)	2.73 (1.57–4.66)	0.66 (0.44–1.52)	1.24 (0.83–2.14)	0.57 (0.10–1.02)
Interquartile range	2.39–4.96	0.35–1.80	0.87–6.04	0.10–1.27	1.52–5.39	0.35–1.80	0.80–2.46	0.10–1.27
Statistics	U = 50.50; *P* < 0.0001		U = 81.50; *P* = 0.0004		U = 128,00; *P* = 0.0002		U = 182.00; *P* = 0.006	

^a^*OSCC* oral squamous cell carcinoma, *CI* confidence interval, *UWS* unstimulated whole saliva, *SWS* stimulated whole saliva.

^b^Number of values below the limit of detection.

No statistically significant difference in MLT levels was obtained between patients with T1N0M0 and T20N0M0 OSCC in UWS (U = 107.00; *P* = 0.248) and SWS (U = 103.00; *P* = 0.195) (Supplement 1).

Median value for serum MLT in OSCC patients (N = 11) was 13.01 (95% CI 10.08–15.14) (Table 2). Median ratios between MLT in UWS and serum MLT and MLT in SWS and serum MLT amounted to 23.66% and 13.15%, respectively.

**Figure 2:**
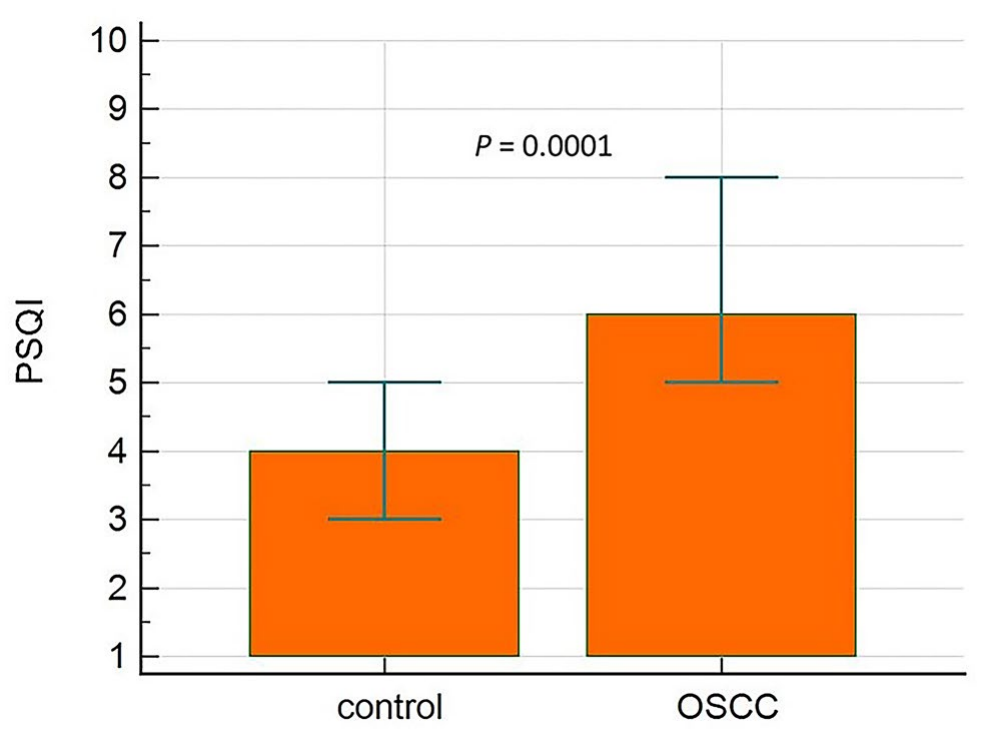
Pittsburgh sleep quality index (PSQI) comparison between the oral squamous cell carcinoma patients (OSCC) and the control group expressed with median and 95% confidence interval for the median.

Sleep quality was significantly lower, i.e. PSQI was significantly higher in OSCC patients than in the control group [median (95% CI) 4 (3–5) vs. 6 (5–8); U = 249.50; *P* = 0.0001] (Fig. 2).

Respondents’ systemic diseases and conditions indexed by ICD-11 and drug consumption indexed by ATC are presented in the Supplement 2 and 3.

**Figure 3:**
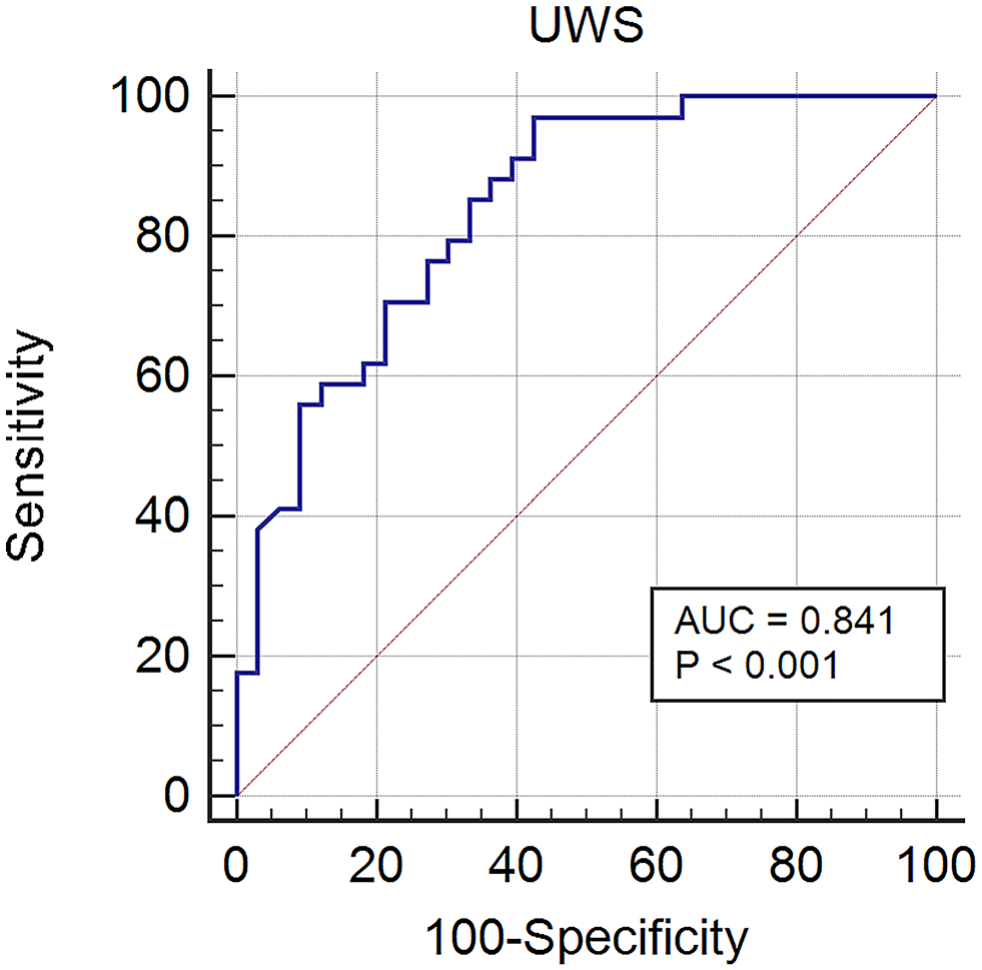
The receiver operating characteristic curve (ROC) for distinguishing patients with oral squamous cell carcinoma from healthy individuals based on values in unstimulated whole saliva (UWS) (*AUC* area under curve; blue line—melatonin (pg/ml); red line—reference line).

ROC analysis was found to be significant (*P* < 0.001) in evaluating the MLT concentration limit in diagnosing OSCC. The area under the curve amounted to 0.84, with a sensitivity of 97.1% (95% CI 84.7–99.9), specificity of 57.6% (95% CI 39.2–74.5) and the MLT concentration limit in UWS of 0.835 pg/ml (Youden Index: 0.546) (Fig. 3). ROC analysis for distinguishing patients with OSCC from healthy individuals based on values in SWS is presented in Supplement 4. ROC analyses for distinguishing patients with T1N0M0 and T2N0M0 OSCC from healthy individuals by MLT levels in UWS are presented in the Supplement 5–8. Comparison of OSCC patients and the control group determined by the cut off values of MLT levels in UWS (Fig. 3) and SWS (Supplement 4) is presented in Supplement 9 and 10, and found statistically significant (Fisher exact test, P < 0.001 and P < 0.001 respectively).

## Discussion

Significantly higher salivary MLT levels were registered in OSCC patients than in healthy subjects. Till this day, not all sources of MLT in saliva are known. Potential synthesis inside the salivary glands has been hypothesized by several authors^30,31^. The concentration of salivary MLT in healthy individuals varies from 1 to 5 pg/ml during daytime and from 10 to 50 pg/ml during nightime^32,33^. Approximately 70% of serum MLT is partially bound to serum albumin^32,33^. It is considered that only unbounded MLT enters the saliva by passive diffusion from serum to the salivary glands, where the MLT concentration reaches up to 33% of the value of MLT serum concentration^33^. Several papers revealed a receptor-dependant transport and storage of MLT inside the parotid glands^30,34^.

Studies on melatonin receptors 1A (MTNR1A) and squamous cell carcinomas in vitro had revealed diminished or even non-existent expression of these receptors due to DNA methylation^35^. Nakamura et al. have suggested MTNR1A as a target for epigenetic silencing at loci 4q35 which may present one of the key events in oral cancerogenesis^36^. In vitro, cessation of squamous cell carcinoma growth that lacked the expression of MTNR1A was achieved by exogenous restoration of MTNR1A receptors^36^.

A possible explanation for elevated salivary MLT levels in OSCC patients could be the MLT receptors disorder in OSCC tissue and thereby the insensitivity of OSCC cells to MLT. This hypothesis could imply the protective direct effect of MLT on healthy oral mucosa, MLT overexpression, insensitivity or decreased expression of MTNR1A, but also the possibility of a yet unknown signal pathway. However, research on the expression of MLT receptors in vivo in OSCC tissue and clinically unchanged oral mucosa tissue in individuals with OSCC is called for to approve or disapprove these hypotheses.

Higher MLT values were registered in UWS than in SWS in both groups (Table 2) and thereby UWS could be considered as more representable for research on salivary MLT levels. We are unfamiliar with the reason for higher MLT levels obtained in UWS than in SWS, however it is established that over 65% of UWS is composed from the submandibular gland saliva and only 20% of the parotic saliva. Stimulated whole saliva is mostly composed from serous parotid gland saliva (> 50%)^37^. Therefore, we can hypothesize higher MLT concentrations in the submandibular than in the parotic saliva.

Serum samples were taken to determine the relationship between the serum and salivary MLT levels in OSCC patients and to compare the serum MLT levels in OSCC patients with the reference serum MLT levels in healthy population available in literature. Median ratios between MLT in UWS and serum MLT and MLT in SWS and serum MLT amounted to 23.66% and 13.15%, respectively. These results are consistent with the expected deviation between serum and salivary MLT levels in healthy individuals and do not support or disapprove the hypothesis of MLT synthesis in salivary glands. Serum MLT levels in healthy individuals start to increase between 6 and 8 p.m. and reach the highest values between midnight and 5 a.m., after which they begin to decrease significantly^38,39^. Between 7 and 9 a.m. MLT levels in serum amount from 7 to 20 pg/ml^38–41^. Those values do not statistically differ from our measurement of serum MLT levels in OSCC patients (median (95% CI): 13.01 pg/ml (10.08–15.14), suggesting that serum MLT concentration may not discriminate patients with early stages OSCC. Obtained interquartile ranges of serum MLT concentrations overlap with those obtained by Stanciu et al. (18.2 (11.0–39.2)^27^. Furthermore, Stanciu et al. suggested that serum MLT concentrations may discriminate patients with OSCC with T-DOI II (depth of invasion) (T > 4 cm; DOI > 10 mm) from healthy controls, due to observed high serum MLT levels obtained in the healthy control group (median 47.6 pg/ml; interquartile range 37.7–66.4; age 57 ± 7 years of age). The serum MLT values below 38.9 pg/ml sampled at 7 a.m. were furthermore identified as values with higher risk for OSCC incidence, with a specificity of 75% and sensitivity of 76.6%. Their measurements, however, are inconsistent with other studies on serum MLT levels with healthy subjects older than fifty^38,39^ and rather correlate with the nocturnal serum levels of MLT in healthy subjects^40,42–44^.

As expected, the PSQI was significantly higher in OSCC patients than in the control group [median (95% CI) 4 (3–5) vs. 6 (5–8); U = 249.50, *P* = 0.0001]. High PSQI did not correlate with serum and salivary MLT values, i.e. we expected lower MLT values in individuals with poor sleep quality, as is the case with some other malignancies^11–13^. The results of this study cannot justify or refute the exogenous MLT intake for sleep improvement. A bigger sample could provide more information on the connection between serum MLT levels and sleep quality in OSCC patients. Given that the salivary MLT concentrations were unknown in OSCC patients, it would not have been a mistake to include subjects with metastatic disease and not only T1N0M0 and T2N0M0 OSCC. However, stratification certainly contributed to the uniformity of this research and could pose potential relevance for future research on this topic. Stanciu et al. obtained statistically significant difference between OSCC patients with and without nodal metastasis^27^. Patients with nodal metastasis had lower MLT values than the ones without nodal metastasis. The authors explained this result by the endocrine involvement in the OSCC progression, possibly suggesting the disturbed circadian MLT rhythm in patients with nodal metastasis. We found no statistically significant difference in salivary MLT levels in UWS and SWS between patients with T1N0M0 and T2N0M0 OSCC, but it is noticeable that slightly higher levels of MLT, both in UWS [median (95% CI) 3.67 (2.36–5.29) vs. 2.73 (1.57–4.66)) and SWS (median (95% CI): 2.42 (0.86–6.22) vs. 1.24 (0.83–2.14)], were detected in T1N0M0 patients. Stanciu et al. also detected higher serum MLT values in T-DOI I (T ≤ 4 cm; DOI ≤ 10 mm) than in T-DOI II (T > 4 cm; DOI > 10 mm)^27^. It would thereby be intriguing to investigate and compare salivary MLT levels in patients with potentially malignant oral disorders and OSCC with and without metastatic disease. ROC analysis was performed and found significant (*P* < 0.001) for the assessment of the salivary MLT concentration limit in OSCC diagnosis. The area under the curve (AUC) amounted 0.84 and the sensitivity of 97.1% and specificity of 57.6% were obtained with an MLT concentration cut-off in the UWS of 0.83 (Youden index: 0.55). The results obtained with ROC analysis are even more representative than some of the presented cumulative biomarkers for OSCC, such as CEA (carcinoembryonic antigen), SCCA (squamous cell carcinoma antigen) and IAP (immunosuppressive acidic protein)^45^.

Given the AUC value, salivary MLT could present a satisfactory diagnostic tool for OSCC as a tumour biomarker alone or in along with some other molecules, such as kininogen 1, cathepsin V, kallikrein 5 or matrix metalloproteinase 1^46–48^. However, larger prospective studies are needed to evaluate the clinical use of salivary MLT as an OSCC biomarker.

This study has several limitations. Due to the sample size, the correlation between alcohol consumption, smoking, particular systemic disease or a drug and the salivary MLT levels could not have been adequately assessed. Thereby, the aforementioned could represent potential confounders. Even though squamous cell carcinomas located on the tongue root, epiglottis or oropharynx were not included in this study, which are more commonly associated with HPV infection, unknown HPV status could present a confounder. The debate in literature on whether HPV infections have the same role in OSCC as in oropharyngeal squamous cell carcinoma is still active. PSQI questionnaire has its drawbacks, as does every survey: the inability to create a fully credible clinical picture, questionable credibility of the testimony and the recall bias.

Determining salivary MLT using ELISA surely has its’ disadvantages, as the possible effect of the plasma constituents from the crevicular fluid and numerous other factors that can influence on the dynamics of serum melatonin levels, such as alcohol and nonsteroidal anti-inflammatory drugs (NSAID) consumption, light exposure, sleep quality and other. Although it is difficult to achieve completely objective preconditions for adequate determination of salivary MLT using ELISA, especially in the single time point, the authors believe that the experimental design and the inclusion criteria for both groups were appropriately rigorous. Mistakes during ELISA sampling are common, but trackable, as the testing is performed in duplicate. High viscosity of certain salivary samples and its rheological properties can present a problem when analysing samples, even after centrifugation, when it is necessary to separate the supernatant from the precipitate. This technical research problem was solved by repeated centrifugation under the same conditions.

We used ROC analysis to evaluate the cut off values of MLT for OSCC diagnosis and to calculate sensitivity and specificity. However, as this is a cross-sectional study and as we have not evaluated patients’ survival time, prognostic value of MLT in OSCC patients could not have been determined. Therefore, this presents a study limitation.

In conclusion, this study revealed elevated salivary MLT levels in patients with T1N0M0 and T2N0M0 OSCC compared to healthy subjects. Sleep quality in OSCC patients was worse than in the control group, however, the expected correlation between salivary MLT levels and sleep quality was not observed in these patients.

## Methods

### Subjects and samples

A cross-sectional study was conducted that involved the experimental (OSCC group) and the control group. Experimental group consisted of patients with histologically verified T1N0M0 and T2N0M0 OSCC, in accordance with the 8th edition of the American Joint Cancer Committee on oral cancer staging^49^, recruited from the Department of Oral and Maxillofacial Surgery, University Hospital Dubrava from January 2017 till November 2019. Respondents with OSCC located on the tongue root and epiglottis were not included in this study. Biopsy was performed at least two weeks before saliva and serum sampling. None of the subjects from the experimental group had received any kind of treatment prior to saliva and serum sampling.

Control group consisted of healthy sex and age matched subjects that attended the Department for a routine examination or a tooth extraction. None of the OSCC patients were hospitalized prior to sampling.

Inclusion criteria for both groups were:

- Abstinence from food and beverages at least 8 h before sampling
- Subjects did not take any NSAID at least 24 h before sampling^50^
- Subjects did not brush their teeth or rinse their mouths with a mouthwash at least 1 h before sampling
- Subjects did not consume alcoholic beverages at least 24 h prior to sampling^51^
- Absence of salivary, jaw and oral mucosal tissue diseases and conditions
- No history of radiation therapy of the head and neck

### Saliva and serum sampling

Saliva and serum were sampled before any surgical procedure between 7 and 9 a.m. in a dark room (< 3 lx). All samples were obtained in the same conditions and by using the specially designed saliva collecting apparatus^52^. Unstimulated and stimulated saliva samples were obtained from every subject. Saliva stimulation was performed by chewing the paraffin blocks. Every saliva sample was stored at – 80 °C until the Enzyme-Linked Immuno-Sorbent Assay (ELISA) testing. The processing of frozen saliva samples and further handling followed the instructions of the ELISA kit manufacturer (IBL International GmbH, Hamburg, Germany). ELISA analysis was performed at the Department of Chemistry and Biochemistry at the University of Zagreb, School of Medicine, from December 2017 till December 2019.

Manufacturer’s ELISA MLT saliva kits had a limit of detection of 0.3 pg/ml. For purpose of taking every value into account, all values below the limit of detection were registered as 0.1 pg/ml.

A blood sample was taken from OSCC patients during routine blood sampling, immediately after saliva sampling. Blood specimens were centrifuged and afterwards stored at -80˚C until ELISA analysis (IBL International GmbH, Hamburg, Germany).

### Indices, medical and dental history

Medical and dental history was taken before sampling. Every subject filled out a Croatian validated version of the Pittsburgh Sleep Quality Index Questionnaire before sampling^53^.

Drug consumption was indexed by the Anatomical Therapeutic Chemical Classification System (ATC)^54^, while the systemic conditions were indexed by the International Statistical Classification of Diseases and Related Health Problems 11 (ICD-11)^55^.

OSCC risk habits were classified as alcohol consumption, tobacco consumption, alcohol and tobacco consumption and as no risk habits. Alcohol consumption was registered as the average daily alcohol consumption expressed in alcohol units. One alcohol unit (a.u.) consisted of 100 ml of wine, 330 ml of beer or 50 ml of hard liquor. Tobacco consumption was registered as the average number of daily smoked cigarettes.

### Statistical analysis

Categorical data are shown by frequency and relative frequency and compared using the χ^2^ test. The post-hoc analysis for χ^2^ test was performed using the t test for proportions. Quantitative data are presented as mean and standard deviation and tested with t test for two groups. Average values of variables that have non-normal distribution (tested with Kolmogorov–Smirnov test) are presented with median and interquartile range, and compared using Mann Whitney's U test. Receiver operating characteristic (ROC) was used to evaluate the cut off values of MLT and to calculate sensitivity and specificity. Fisher exact test was used to compare the number of patients in the experimental and control group correctly identified with the cut off values of MLT in UWS and SWS. The correlation was calculated by Spearman's nonparametric correlation coefficient. Sample size calculation for testing the hypothesis was based on the results of the MLT circadian values^32,37,38^. The significant sample size was calculated using the G Power 3.1.9.2^56^. Given that the salivary MLT levels in healthy individuals amount from 1 to 5 pg/ml between 7 and 9 a.m.^32^, we expected salivary MLT levels in OSCC patients bellow 0.8 pg/ml. With an α = 0.05 and the power of 0.80 the suggested sample size was 12 subjects. Serum MLT levels of healthy individuals over 40 years age amount between 7 and 20 pg/ml between 7 and 9 a.m.^37,38^, thereby values bellow 5 pg/ml were expected in OSCC patients. With an α = 0.05 and the power of 0.80 the suggested sample size was 9 subjects. Data was collected and stored to the database in MS Excel. Statistical data processing was done using the MedCalc ver. 16.2.1. (MedCalc Software, Ostend, Belgium). The level of statistical significance was set at 5% (*P* < 0.05) and all confidence intervals were given at a 95% level.

The study was approved by the Ethics Committee of the School of Dental Medicine, University of Zagreb and the University Hospital Dubrava Ethics Committee. All methods were performed in accordance with the relevant guidelines and regulations. Written informed consent was obtained from all participants. This manuscript was written in accordance with the STROBE recommendations.

## Supplementary Information

Supplementary Information 1.

## Data Availability

The datasets used and/or analysed during the current study are available from the corresponding author on reasonable request.

## References

[1] Lee YA (2019). Tobacco smoking, alcohol drinking, betel quid chewing, and the risk of head and neck cancer in an East Asian population. Head Neck..

[2] Leonel ACLDS, Soares CBRB, Lisboa de Castro JF, Bonan PRF, Ramos-Perez FMM, Perez DEDC (2019). Knowledge and attitudes of primary health care dentists regarding oral cancer in Brazil. Acta Stomatol. Croat..

[3] Kaskas MN, Moore-Medlin T, McClure GB, Ekshyyan O, Vanchiere JA, Nathan CAO (2014). Serum biomarkers in head and neck squamous cell cancer. JAMA Otolaryngol. Head Neck Surg..

[4] Khurshid Z, Zafar MS, Khan RS, Najeeb S, Slowey PD, Rehman IU (2018). Role of salivary biomarkers in oral cancer detection. Adv. Clin. Chem..

[5] Santosh ABR, Jones T, Harvey J (2016). A review on oral cancer biomarkers: Understanding the past and learning from the present. J. Cancer Res. Ther..

[6] D’souza S, Addepalli V (2018). Preventive measures in oral cancer: An overview. Biomed. Pharmacother..

[7] Moradkhani F, Moloudizargari M, Fallah M, Asghari N, HeidariKhoei H, Asghari MH (2020). Immunoregulatory role of melatonin in cancer. J. Cell Physiol..

[8] Luo J (2020). Effect of melatonin on T/B cell activation and immune regulation in pinealectomy mice. Life Sci..

[9] Elsabagh HH, Moussa E, Mahmoud SA, Elsaka RO, Abdelrahman H (2020). Efficacy of Melatonin in prevention of radiation-induced oral mucositis: A randomized clinical trial. Oral Dis..

[10] Pazarci P (2020). The effects of daylight exposure on melatonin levels, Kiss1 expression, and melanoma formation in mice. Croat Med. J..

[11] Yang WS, Deng Q, Fan WY, Wang WY, Wang X (2014). Light exposure at night, sleep duration, melatonin and breast cancer: A dose-response analysis of observational studies. Eur. J. Cancer Prev..

[12] Zhao M (2016). The reduction in circulating melatonin level may contribute to the pathogenesis of ovarian cancer: A retrospective study. J. Cancer..

[13] Chen D, Yin Z, Fang B (2018). Measurements and status of sleep quality in patients with cancers. Support Care Cancer.

[14] Lissoni P (1999). Decreased toxicity and increased efficacy of cancer chemotherapy using the pineal hormone melatonin in metastatic solid tumour patients with poor clinical status. Eur. J. Cancer.

[15] Lin PH (2020). Melatonin activates cell death programs for the suppression of uterine leiomyoma cell proliferation. J. Pineal Res..

[16] Chen Y, Zhang T, Liu X, Li Z, Zhou D, Xu W (2018). Combination of melatonin and rapamycin for head and neck cancer therapy: Suppression of AKT/mTOR pathway activation, and activation of mitophagy and apoptosis via mitochondrial function regulation. J. Pineal Res..

[17] Kim TW, Jeong JH, Hong SC (2015). The impact of sleep and circadian disturbance on hormones and metabolism. Int. J. Endocrinol..

[18] Chang WP, Lin CC (2017). Relationships of salivary cortisol and melatonin rhythms to sleep quality, emotion, and fatigue levels in patients with newly diagnosed lung cancer. Eur. J. Oncol. Nurs..

[19] Innominato PF (2016). The effect of melatonin on sleep and quality of life in patients with advanced breast cancer. Support Care Cancer.

[20] Rafie C, Ning Y, Wang A, Gao X, Houlihan R (2018). Impact of physical activity and sleep quality on quality of life of rural residents with and without a history of cancer: Findings of the Day and Night Study. Cancer Manag. Res..

[21] Tarquini R (1995). Serum melatonin in multiple myeloma: Natural brake or epiphenomenon?. Anticancer Res..

[22] Vician M, Zeman M, Herichová I, Juráni M, Blazícek P, Matis P (1999). Melatonin content in plasma and large intestine of patients with colorectal carcinoma before and after surgery. J. Pineal Res..

[23] Feuer GM, Kerenyi NA (1989). Role of the pineal gland in the development of malignant melanoma. Neurochem. Int..

[24] Lu H (2017). Melatonin represses oral squamous cell carcinoma metastasis by inhibiting tumor-associated neutrophils. Am. J. Transl. Res..

[25] Yeh CM, Lin CW, Yang JS, Yang WE, Su SC, Yang SF (2016). Melatonin inhibits TPA-induced oral cancer cell migration by suppressing matrix metalloproteinase-9 activation through the histone acetylation. Oncotarget.

[26] Yang CY (2017). Melatonin exerts anti-oral cancer effect via suppressing LSD1 in patient-derived tumor xenograft models. Oncotarget.

[27] Stanciu AE (2020). Clinical significance of serum melatonin in predicting the severity of oral squamous cell carcinoma. Oncol. Lett..

[28] Kaczor-Urbanowicz KE, Carreras-Presas CM, Aro K, Tu M, Garcia-Godoy F, Wong DTW (2017). Saliva diagnostics—current views and directions. Exp. Biol. Med..

[29] Gleber-Netto FO (2016). Salivary biomarkers for detection of oral squamous cell carcinoma in a Taiwanese population. Clin. Cancer Res..

[30] Isola M, Lilliu MA (2016). Melatonin localization in human salivary glands. J. Oral. Pathol. Med..

[31] Shimozuma M (2011). Expression and cellular localizaion of melatonin-synthesizing enzymes in rat and human salivary glands. Histochem. Cell Biol..

[32] van Faassen M, Bischoff R, Kema IP (2017). Relationship between plasma and salivary melatonin and cortisol investigated by LC-MS/MS. Clin. Chem. Lab. Med..

[33] Laakso ML, Porkka-Heiskanen T, Alila A, Stenberg D, Johansson G (1990). Correlation between salivary and serum melatonin: Dependence on serum melatonin levels. J. Pineal Res..

[34] Isola M (2013). Subcellular distribution of melatonin receptors in human parotid glands. J. Anat..

[35] Gómez-Moreno G, Guardia J, Ferrera MJ, Cutando A, Reiter RJ (2010). Melatonin in diseases of the oral cavity. Oral Dis..

[36] Nakamura E (2008). Frequent silencing of a putative tumor suppressor gene melatonin receptor 1 A (MTNR1A) in oral squamous-cell carcinoma. Cancer Sci..

[37] Humphrey SP, Wiliamson RT (2001). A review of saliva: Normal composition, flow, and function. J. Prostheth. Dent..

[38] Tozawa T, Mishima K, Satoh K, Echizenya M, Shimizu T, Hishikawa Y (2003). Stability of sleep timing against the melatonin secretion rhythm with advancing age: Clinical implications. J. Clin. Endocrinol. Metab..

[39] Nogueira LM (2013). Individual variations in serum melatonin levels through time: Implications for epidemiologic studies. PLoS One.

[40] Karasek M, Kowalski AJ, Suzin J, Zylinska K, Swietoslawski J (2005). Serum melatonin circadian profiles in women suffering from cervical cancer. J. Pineal Res..

[41] Scholtens RM, van Munster BC, van Kempen MF, de Rooij SE (2016). Physiological melatonin levels in healthy older people: A systematic review. J. Psychosom. Res..

[42] Khaleghipour S (2012). Morning and nocturnal serum melatonin rhythm levels in patients with major depressive disorder: An analytical cross-sectional study. Sao Paulo Med. J..

[43] Fatima G, Sharma VP, Verma NS (2016). Circadian variations in melatonin and cortisol in patients with cervical spinal cord injury. Spinal Cord.

[44] Sutherland ER, Ellison MC, Kraft M, Martin RJ (2003). Elevated serum melatonin is associated with the nocturnal worsening of asthma. J. Allergy Clin. Immunol..

[45] Kurokawa H, Tsuru S, Okada M, Nakamura T, Kajiyama M (1993). Evaluation of tumor markers in patients with squamous cell carcinoma in the oral cavity. Int. J. Oral Maxillofac. Surg..

[46] Yu JS (2016). Saliva protein biomarkers to detect oral squamous cell carcinoma in a high-risk population in Taiwan. Proc. Natl. Acad. Sci. USA.

[47] Feng Y (2019). Salivary protease spectrum biomarkers of oral cancer. Int. J. Oral Sci..

[48] Amiri Dash Atan N, Koushki M, Rezaei Tavirani M, Ahmadi NA (2018). Protein–protein interaction network analysis of salivary proteomic data in oral cancer cases. Asian Pac. J. Cancer Prev..

[49] Amin MB (2017). AJCC Cancer Staging Manual.

[50] Murphy PJ, Myers BL, Badia P (1996). Nonsteroidal anti-inflammatory drugs alter body temperature and suppress melatonin in humans. Physiol. Behav..

[51] Maldonado MD, Moreno H, Calvo JR (2009). Melatonin present in beer contributes to increase the levels of melatonin and antioxidant capacity of the human serum. Clin. Nutr..

[52] Salarić I, Sabalić M, Alajbeg I (2017). Opiorphin in burning mouth syndrome patients: A case–control study. Clin. Oral Investig..

[53] Lušić L, Pecotić R, Valić M, Pavlinac Dodig I, Đogaš Z (2014). Sleep Quality and Other Psychological Variables in Obstructive Sleep Apnea Patients. The Oxford Sleep and Circadian Neuroscience Summer Schools.

[54] World Health Oragnization Collaborating Centre for Drug Statistics Methodology. WHOCC-ATC/DDD Index. Preprint at https://www.whocc.no/atc_ddd_index/(2015).

[55] World Health Oragnization. International Statistical Classification of Diseases and Related Health Problems (ICD-11). https://www.who.int/classifications/icd/en/(2018).

[56] Faul F, Erdfelder E, Buchner A, Lang AG (2009). Statistical power analyses using G*Power 3.1 Statistical power analyses using G*Power 3.1: Tests for correlation and regression analyses. Behav. Res. Methods.

