# Supplementary material for: Salivary melatonin in oral squamous cell carcinoma patients
Source: Sci Rep. 2021 Jun 24;11:13201. doi: 10.1038/s41598-021-92649-3 (PMC8225878; doi:10.1038/s41598-021-92649-3)
Supplement: Supplementary file 1 — Supplementary Information 1. [file 41598_2021_92649_MOESM1_ESM.docx]

Salivary melatonin in oral squamous cell carcinoma patients

Ivan Salarić, Ivana Karmelić, Jasna Lovrić, Ksenija Baždarić, Marko Rožman, Igor Čvrljević, Ivan Zajc, Davor Brajdić, Darko Macan

Supplement 1. Comparison of salivary melatonin levels (pg/ml) in unstimulated whole saliva (UWS) and stimulated whole saliva (SWS) between the oral squamous cell carcinoma (OSCC) subgroups (T1N0M0 and T2N0M0) expressed with median and 95% confidence interval for the median.


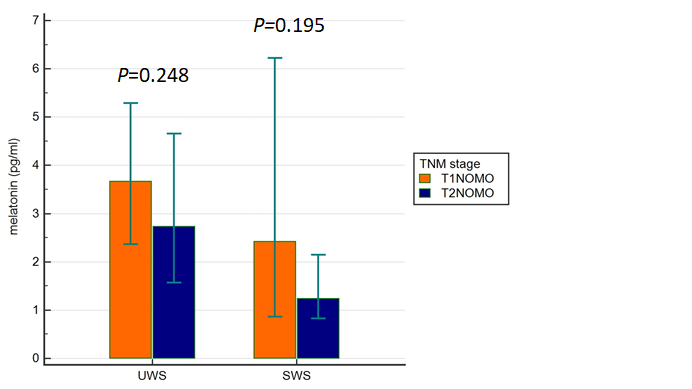


Supplement 2. Respondents’ systemic conditions and diseases classified using the International Classification of Diseases 11th Revision (ICD -11)

| Systemic condition or disease (ICD-11) | OSCC group (N) | Control group (N) | Total (N) |
| --- | --- | --- | --- |
| Healthy / no systemic conditions | / | 26 | 26 |
| Hypertension (I10) | 10 | 6 | 16 |
| Atherosclerosis (I70) | 6 | 2 | 8 |
| Hyperlipidaemia (5C80Z) | 7 | 3 | 10 |
| Phlebitis and trombophlebitis (I80) | 2 | 0 | 0 |
| Angina pectoris (I20) | 2 | 1 | 3 |
| Varicose veins of the lower extremities (I83) | 0 | 2 | 0 |
| Gastroesophageal reflux disease (DA22) | 9 | 0 | 9 |
| Anxiety (MB24.3) | 7 | 0 | 7 |
| Hyperthyreodism (5A02) | 0 | 1 | 1 |
| Alcohol-induced psychotic disorder (6C40.6) | 3 | 0 | 3 |
| Psychosis (6A2Y) | 0 | 1 | 0 |
| Chronic kidney disease (GB61) | 1 | 0 | 1 |
| Deep vein thrombosis (BD71) | 1 | 0 | 1 |
| Calculus of kidney (GB70.0Z) | 2 | 0 | 2 |
| Hypokalaemia (5C77) | 3 | 0 | 3 |
| Osteoporosis (M81) | 1 | 0 | 1 |
| Depression (F32) | 3 | 1 | 4 |
| Prostatic hypertrophy (GA90) | 4 | 2 | 6 |
| Type 2 diabetes mellitus (E11) | 2 | 0 | 0 |
| Asthma (J45) | 3 | 0 | 3 |

* OSCC = oral squamous cell carcinoma

Supplement 3. Respondents' drug consumption classified using the Anatomical Therapeutic Chemical (ATC) Classification System

| Drug (ATC) | OSCC group (N) | Control group (N) | Total (N) |
| --- | --- | --- | --- |
| Do not take any medication | 10 | 26 | 36 |
| Calcium inhibitors (C08) | 0 | 2 | 2 |
| Proton pump inhibitors (A02BC) | 6 | 0 | 6 |
| Acetylsalicylic acid (B01AC30) | 3 | 1 | 4 |
| Benzodiazepine derivatives (N05CD) | 7 | 2 | 9 |
| Thiouracils (H03BA) | 0 | 1 | 1 |
| Alpha-adrenoreceptor antagonists (C02CA) | 1 | 1 | 2 |
| Angiotensin II receptor blockers, plain (C09CA) | 2 | 0 | 2 |
| Sulfonamides, plain (C03CA) | 6 | 1 | 7 |
| Beta blocking agents, selective (C07AB) | 10 | 1 | 11 |
| Selective beta-2-adrenoreceptor agonists (R03AC) | 3 | 0 | 3 |
| Alpha-adrenoreceptor antagonists (G04CA) | 4 | 2 | 6 |
| Clindamycin (G01A10) | 1 | 1 | 2 |
| Vitamin D and analougs (A11CC) | 1 | 0 | 1 |
| Antipsychotics (N05A) | 3 | 1 | 4 |
| ACE inhibitors, plain (C09AA) | 8 | 4 | 12 |
| HMG CoA reductase inhibitors (C10AA) | 7 | 3 | 10 |
| Heparin (B01AB) | 6 | 0 | 6 |
| Opioids (N02A) | 2 | 0 | 2 |
| Blood glucose lowering drugs, excl. insulins (A10B) | 2 | 0 | 2 |
| Potassium (A12BA30) | 3 | 0 | 3 |
| H2-receptor antagonists (A02BA) | 5 | 0 | 5 |
| Antigout preparations (M04A) | 1 | 0 | 1 |
| Calcium acetate and magnesium carbonate (V03AE04) | 1 | 0 | 1 |
| Warfarin (B01AA03) | 1 | 0 | 1 |
| Vasodilators used in cardiac diseases (C01D) | 2 | 1 | 0 |
| Trimetadizine (C01EB15) | 1 | 0 | 1 |
| Antidepressants (N06A) | 3 | 1 | 4 |

* OSCC = oral squamous cell carcinoma

Supplement 4. The receiver operating characteristic curve (ROC) for distinguishing patients with oral squamous cell carcinoma from healthy individuals based on values in stimulated whole saliva (SWS). The area under the curve amounted to 0.765, with a sensitivity of 85.29% (95% CI: 68.9–95.0),specificity of 60.61% (95% CI: 42.1–77.1) and the melatonin concentration limit in SWS of 0.704 pg/ml (Youden Index: 0.459).

Supplement 5. The receiver operating characteristic curve for distinguishing T1N0M0 oral squamous cell carcinoma patients from healthy control group based on values in unstimulated whole saliva (UWS). The area under the curve amounted to 0.891, with a sensitivity of 92.86% (95% CI: 66.1-99.9), specificity of 72.73% (95% CI: 54.5-86.7) and the melatonin concentration limit in UWS of 1.689 pg/ml (Youden Index: 0.656).

Supplement 6. The receiver operating characteristic curve for distinguishing T2N0M0 oral squamous cell carcinoma patients from healthy control group based on values in unstimulated whole saliva (UWS). The area under the curve amounted to 0.806, with a sensitivity of 95.00% (95% CI: 75.1 – 99.9), specificity of 57.58% (95% CI: 39.2 – 74.5) and the melatonin concentration limit in UWS of 0.835 pg/ml (Youden Index: 0.526).

Supplement 7. The receiver operating characteristic curve for distinguishing T1N0M0 oral squamous cell carcinoma patients from healthy control group based on values in stimulated whole saliva (SWS). The area under the curve amounted to 0.824, with a sensitivity of 71.43% (95% CI: 41.9 – 91.6), specificity of 84.85% (95% CI: 68.1 – 94.9) and the melatonin concentration limit in SWS of 1.425 pg/ml (Youden Index: 0.563).

Supplement 8. The receiver operating characteristic curve for distinguishing T2N0M0 oral squamous cell carcinoma patients from healthy control group based on values in stimulated whole saliva (SWS). The area under the curve amounted to 0.724, with a sensitivity of 85.00% (95% CI: 62.1 – 96.8), specificity of 60.61% (95% CI: 42,1 - 77,1) and the melatonin concentration limit in SWS of 0.704 pg/ml (Youden Index: 0.456).

Supplement 9. Comparison of OSCC patients and control group determined by the cut off value (0.835 pg/ml; Figure 3) of melatonin in unstimulated whole saliva

| UWS (pg/ml) | <0.834  N (%) | ≥ 0.835  N(%) | Total  (N (%) |  |  | P* |  |
| --- | --- | --- | --- | --- | --- | --- | --- |
| Control group (N, %^§^) | 19 (57.6) | 14 (42.4) | 33 (49.3%) |  |  | <.0.001 |  |
| OSCC group (N,% ^§^) | 1 (2.9) | 33 (97.1) | 34 (50.7%) |  |  |  |  |
| Total (N, %) | 20 (29.9%) | 47 (70.1%) | 67 |  |  |  |  |

* Fisher exact test; ^§^ Row total percentage; UWS = unstimulated whole saliva; OSCC = oral squamous cell carcinoma

Supplement 10. Comparison of OSCC patients and control group determined by the cut off value (0.704 pg/ml; Supplement 4) of melatonin in stimulated whole saliva

| SWS (pg/ml) | <0.703  N(%) | ≥ 0.704  N(%) | Total  (N, %) |  |  | P* |  |
| --- | --- | --- | --- | --- | --- | --- | --- |
| Control group (N, %*) | 20 (60.6) | 13 (39.4) | 33 (49.3%) |  |  | <.0.001 |  |
| OSCC group (N, %*) | 5 (14.7) | 29 (85.3) | 34 (50.7%) |  |  |  |  |
| Total (N, %) | 25 (37.3%) | 42 (62.7%) | 67 |  |  |  |  |

* Fisher exact test; ^§^ Row total percentage; SWS = stimulated whole saliva; OSCC = oral squamous cell carcinoma
